# ‘We are all in the same boat’: a qualitative cross-sectional analysis of COVID-19 pandemic imagery in scientific literature and its use for people working in the German healthcare sector

**DOI:** 10.3389/fpsyt.2024.1296613

**Published:** 2024-02-05

**Authors:** Andreas M. Baranowski, Rebecca Blank, Katja Maus, Simone C. Tüttenberg, Julia-K. Matthias, Anna C. Culmann, Lukas Radbruch, Cornelia Richter, Franziska Geiser

**Affiliations:** ^1^Department of Psychosomatic Medicine and Psychotherapy, University Hospital Bonn, University of Bonn, Bonn, Germany; ^2^Systematic Theology and Hermeneutics, Faculty of Protestant Theology, University of Bonn, Bonn, Germany; ^3^Department of Palliative Medicine, University Hospital Bonn, University of Bonn, Bonn, Germany

**Keywords:** COVID-19 pandemic, imagery, HCW, healthcare, metaphor, resilience

## Abstract

**Background:**

The COVID-19 pandemic presents a significant challenge to professional responders in healthcare settings. This is reflected in the language used to describe the pandemic in the professional literature of the respective professions. The aim of this multidisciplinary study was to analyze the linguistic imagery in the relevant professional literature and to determine the identification of different professional groups with it and its emotional effects.

**Method:**

A list of 14 typical, widespread and differing imageries for COVID-19 in form of single sentences (e.g., “Until the pandemic is over, we can only run on sight.”) were presented to 1,795 healthcare professionals in an online survey. The imageries had been extracted from a qualitative search in more than 3,500 international professional journals in medicine, psychology and theology. Ratings of agreement with these imageries and feelings about them were subjected to factor analysis.

**Results:**

Based on the list of imageries presented, it was possible to identify three factors for high/low agreement by experiences, and two factors for high/low induced feelings. Broad agreement emerged for imageries on “fight against the crisis” and “lessons learned from the crisis”, while imageries on “acceptance of uncontrollability” tended to be rejected. Imageries of “challenges” tended to lead to a sense of empowerment among subjects, while imageries of “humility” tended to lead to a sense of helplessness.

**Conclusion:**

Based on the qualitative and subsequential quantitative analysis, several factors for imageries for the COVID-19 pandemic were identified that have been used in the literature. Agreement with imageries is mixed, as is the assessment of how helpful they are.

## Introduction

The COVID-19 pandemic constituted a significant challenge to the global healthcare system. For instance, employees in the healthcare system reported substantial amounts of psychological distress (1–3), which manifested in symptoms associated with depression, anxiety and post-traumatic stress disorder (4–6).

However, challenges differed across professional groups. While physicians and nurses were faced with particularly difficult working conditions, especially in the beginning of the pandemic, which made this group especially vulnerable to mental distress (7–9), other groups, such as psychologists, were able to reduce patient contact, at least in part, e.g., with the help of telemedicine (10). At the same time, the challenges faced by hospital spiritual/pastoral care workers remains largely unknown. This group plays an important role in the care of elderly and palliative patients (11, 12), but was largely considered dispensable and therefore received little attention in the public discourse of the crisis (13).

The challenges of COVID-19 were also reflected in the language used to describe the pandemic. Linguistic imagery (e.g., metaphors, similes, personifications) was widely used in professional (14–17), political (18–20), and media (18, 21, 22) communication to describe the COVID-19 virus and the difficulties associated with a global pandemic. Imagery relating to struggle and war (e.g., physicians as warriors, virus as enemy) were particularly prevalent (14, 18, 20, 23–25). However, other imagery regarding transformative processes, e.g., “People who have suffered through the crisis are different, than they were before” (17), but also fear and uncertainties, e.g., “It’s like cancer. Because you say I will not catch it, but if you don’t take precautions, it can kill” (15, 26), were also used frequently.

Only few empirical studies have looked at the effects of imagery on the recipient during the COVID-19 pandemic and it is therefore still an open question, how imagery affects the perception of the pandemic. Pisano and colleagues (27) demonstrated that participants had formed new semantic associations (e.g., “trench”—”hospital”) during the pandemic, that were stronger and more readily available than classical associations (e.g., “trench”—”soldier”). Further research showed that, by experimentally creating and comparing different news articles about the pandemic, the inclusion of metaphors in the articles predicted greater self-efficacy in readers (28). This was particularly true for metaphors referring to the possibility of change, but was also found for war metaphors. In line with these findings, Naamati-Schneider and Gabay (16) found that metaphors that created a sense of mission and meaningfulness were helpful in coping with an extreme health crisis, while metaphors that generated a sense of isolation and sacrifice intensified helplessness and fear, thus undermining effective coping mechanisms. Past research has also shown that seriously ill patients found it helpful when healthcare professionals used metaphors in their conversations (29). For a general overview of the use of metaphors in the healthcare sector, see (17).

The aim of this multidisciplinary study was to analyze imagery of the COVID-19 pandemic in the medical, psychological, and theological professional literature, and to determine the feelings of different professional groups towards it. This helps to understand how different professional groups see themselves in the pandemic, but also gives insight into what language is helpful for these groups when talking about the pandemic. Additionally, we wanted to find out how identification with certain linguistic imagery was predictive for stressors of the pandemic, or protective personality traits.

## Materials and methods

### Data collection

The online survey was conducted in March and April 2022. This was at the end of the fifth COVID-19 wave, after two years of pandemic, with many public safety measures starting to loosen up in Germany (30). The participation link was provided through online platforms and mailing lists of a large German university hospital and further general hospitals as well as several medical professional associations. The study was approved by the ethics committee of the local medical faculty (reference number: 125_20). All participants provided their online informed consent prior to completing the survey.

The survey consisted of 137 items and took approximately 30 minutes to complete. The complete questionnaire, including all scales, was administered in German. This included questions regarding age, gender, living conditions, children, migration background, occupational characteristics, profession, years of professional experience, employment status and a number of further questionnaires. In our analysis, we focused on age, gender, profession and the presented questionnaires in measures.

Unipark (www.unipark.com) was used to program and host the survey. Inclusion criteria for participation were a minimum age of 18 years, working in the healthcare sector, residence/working place in Germany, and sufficient German language skills.

### Sample characteristics

A total of 1,795 participants completed the questionnaire and were included in the analysis. The majority of the sample consists of women (*n* = 1,301), with 491 men and three people who identified as diverse. The gender distribution in our sample is representative of the overall gender distribution in the healthcare sector within the population we researched, as well as reflective of global trends in healthcare sector employment (31). The participants who identified themselves as diverse were included in all analyses except for those looking at gender differences, because the sample size was too small for a meaningful analysis. Age was assessed based on 5 groups, with the majority falling in the range of 51-60 (*n* = 504) followed by age 41-50 (*n* = 410), age 31-40 (*n* = 400), age 18-30 (*n* = 331), and age > 60 (*n* = 150). Participants were placed in 5 occupational groups, based on their self-disclosure; physicians (*n* = 330), nurses (*n* = 508), psychologists (*n* = 55), spiritual care workers (*n* = 124) and others (*n* = 778). Spiritual care workers in this sample are primarily Protestant or Catholic theologians with additional training to offer comprehensive spiritual support in hospital settings. Their services include counseling, spiritual guidance, and emotional support for patients and their families, functioning as part of a multidisciplinary healthcare team. Others consisted of a wide range of professions, including e.g., students, administrative staff, physiotherapists, and social workers, and served as a general reference group.

### Measures

#### Imageries

Approximately 3,500 articles from journals in the fields of (a) life sciences, medicine, and healthcare systems, psychology, psychiatry, and the wider mental health system, (b) theology (including Protestant, Catholic, and spiritual care), (c) social sciences and philosophy (including education), and (d) exemplary findings in political and social sciences were searched from 2020 and 2021. This search utilized the databases PubMed, KVK (encompassing all German catalogues, WorldCat, and National Library of Medicine), and Index Theologicus. Search terms used were “COVID-19”, “corona”, and “SARS-CoV-2”, common to both English and German, as well as “crisis” and its German counterparts “Krise” and “Krisenbewältigung”. Each of these terms was paired with “resilience” or “Resilienz” in their respective languages. The results were collected in Citavi (Version 6) and MAXQDA (Version 2020) for which full-text versions were obtainable. The database search was initially conducted using the specified search terms, followed by a detailed review based on the titles, introductions, and conclusions of the articles. The English and German international material proved more heterogeneous than expected in terms of text genres, content, and methodologies used; it included inter-, multidisciplinary, and transdisciplinary research work by internationally assembled research teams. Consequently, we applied hermeneutic methods from the humanities (textual and linguistic analysis of active, passive, mediopassive directions of individual and collective agency, 1^st^ and 3^rd^ person perspectives, temporal dynamics, etc.) and discussed the results in a structured group process. A multidisciplinary consortium of 10 physicians, psychologists, and theologians identified 14 widely used linguistic imageries of the SARS-Cov-2 pandemic, intended to represent as broad a spectrum of the language used as possible (e.g., ‘Until the pandemic is over, we can only run on sight’).

Following this selection process, participants in the study were presented with the sentences and asked how much they agreed with them [not agreeing at all (1) – completely agree (4)] and how they felt based on it [very helpless (1) – very enabled (5)]. These two aspects will be labeled “agreement” and “induced resolve” in the further text. Background for the choice of these questions was the premise that an imagery will have more impact if firstly a person highly agrees or is highly identified with its meaning, and if secondly it helps to mobilize feelings of resolve and control (16, 28). The sentences presented are listed in English translation in **Tables 1**, **2**, the original German wording of the items used in this study can be found in **Supplementary A**.

**Table 1:** Three-factor solution for the scale “agreement”.

Item	Factor		
	1. Fight	2. Lessons	3. Acceptance
The heroes and heroines of the crisis are those who stay at their posts and give their all where few see it.	.709	.087	.055
In the pandemic, nurses are on the front lines for all of us.	.683	.149	.122
In times of the pandemic, it becomes clear that we are all in the same boat and can only get ahead if we row together.	.595	.392	-.030
The virus is an invisible enemy.	.559	-.100	.463
Now we must seize the opportunity to drive digitization forward.	.445	.267	-.062
Our new life begins here and now. Not only after the crisis.	.046	.731	-.001
The pandemic shows that we have to accept that normality means change.	.170	.652	.054
The crisis has reminded many people that they too will die.	.222	.515	.228
The pandemic is the stress test for churches to prove that they recognize what people really need.	.069	.500	.223
Corona teaches us through distance from each other what closeness really means.	.391	.476	.135
Such a small virus manages to create a sense of community, we must ensure that the feeling remains.	.409	.429	.124
It is not in our hands how the crisis will turn out, we can only trust.	-.113	.202	.758
Corona shows that there is nothing you can do about the violent storm; you have to endure it patiently.	.033	.138	.753
Until the pandemic is over, we can only run on sight.	.268	.092	.585

**Table 2:** Two-factor solution for the scale “induced resolve”.

Item	Factor	
	1. Challenges	2. Humility
In times of the pandemic, it becomes clear that we are all in the same boat and can only get ahead if we row together.	.736	.190
The heroes and heroines of the crisis are those who stay at their posts and give their all where few see it.	.698	.162
Our new life begins here and now. Not only after the crisis.	.683	.138
Corona teaches us through distance from each other what closeness really means.	.671	.196
In the pandemic, nurses are on the front lines for all of us.	.646	.231
Now we must seize the opportunity to drive digitization forward.	.633	.026
Such a small virus manages to create a sense of community, we must ensure that the feeling remains.	.629	.224
The pandemic shows that we have to accept that normality means change.	.618	.324
Corona shows that there is nothing you can do about the violent storm; you have to endure it patiently.	.119	.809
It is not in our hands how the crisis will turn out, we can only trust.	.064	.808
The virus is an invisible enemy.	.192	.731
Until the pandemic is over, we can only run on sight.	.198	.679
The crisis has reminded many people that they too will die.	.402	.530
The pandemic is the stress test for churches to prove that they recognize what people really need.	.281	.436

#### Transpersonal trust

The Transpersonal Trust scale (TPV) was used to assess religiosity and spirituality (32). The scale describes a person who recognizes the existence of a higher reality, trusts in it, and experiences a strong connection with it (e.g., “I feel connected to a higher reality/being/God. I can trust in this even in difficult times”) and has been previously employed in studies with healthcare workers during the COVID-19 pandemic (33). It consists of 11 items and is rated on a four-point Likert scale ranging from 0 (“does not apply at all”) to 3 (“applies completely”). In our sample, the TPV demonstrated high reliability with a Cronbach’s α = .84.

#### Depressive and anxiety symptoms (PHQ-4)

Depressive and general anxiety symptoms over the last two weeks were assessed with the Patient Health Questionnaire PHQ-4 (34), which has been used in the studied sample before (35). The questionnaire consists of four items (e.g., “Feeling nervous, anxious or on edge” and “Feeling down, depressed or hopeless”) and is answered on a Likert scale from 0 (“not at all”) to 3 (“almost every day”). Cronbach’s α in this sample is .83.

#### Impact of event scale (IES-6)

The IES-6 is a 6-item short version of the Impact of Event Scale-Revised (IES-R). It measures the principal components of PTSD on a four-point Likert scale from 0 (“not at all”) to 3 (“often”). The instructions were tailored to the coronavirus and questions included “I tried not to think about it” and “I felt watchful or on-guard” (36). This approach has previously been used for studying PTSD in healthcare workers during the COVID-19 pandemic (6). Internal consistency of the IES-6 is Cronbach’s α = .73 in the present study.

#### Optimism

Optimism was assessed based on Kemper et al. (37), using the item “How optimistic are you in general?”, which is answered on a seven-point Likert-scale from 1 (“Not optimistic at all”) to 7 (“very optimistic”). Higher values reflect a higher level of optimism. This question has been deployed to study optimism in healthcare workers during the COVID-19 pandemic before (38).

#### COVID-19-related variables

The questionnaire included a range of COVID-19 related variables. In this analysis, we focused on problems related to COVID-19, which were measured with 18 items on a scale from 0 “strongly disagree” to 4 “strongly agree”, based on Matsuishi et al. (39). Items focused, among other things, on anxiety about infection, sleep problems, physical or mental exhaustion, smoking, and drinking alcohol, during the COVID-19 pandemic over the past 2 weeks and included items such as “I was afraid to become infected” and “I felt physically or mentally exhausted”. Items were deployed before to measure COVID-19-related problems in this population (2) A mean score of all answers was calculated with a Cronbach’s α = .75 in this study.

### Statistical analysis

All statistical analyses were conducted using IBM SPSS Statistics (Version 26) and R (Version 4.1.1). To explore the factor structure in the imageries, all 14 sentences were treated as a scale and a factor analysis with Varimax rotation was run with them. Internal consistency was measured with Cronbach’s α. For descriptive and comparative statistics, analyses of variance (ANOVA) were performed and effect size given in partial η^2^. In case of multiple comparisons, Tukey *post-hoc* tests were conducted and effect size given in Cohen’s *d*.

## Results

### Factor analysis of imageries

A factor analysis was conducted to explore the structure of the imageries for agreement and induced resolve. The objective of the factor analysis was to check whether the imageries could be placed in meaningful factor structures based on these two assessments and use this as a basis for further analysis.

First, we obtained a Kaiser-Meyer-Olkin (KMO) index for agreement and hope of .85 and .91 respectively, with a highly significant Bartlett’s sphericity test for both scales (*p* < .001). The Cattell (40) scree test (Eigenvalues) suggested a three-factor solution for “agreement” and a two-factor solution for “induced resolve” based on the imageries. The three factors explained 45.74% of the total variance for agreement and were named “fight against the crisis”, “lessons from the crisis” and “acceptance of uncontrollability”, based on the included items. The two factors of induced resolve explained 49.85% of the total variance and were named “challenges” and “humility” (see **Tables 1**, **2**).

Internal consistency was Cronbach’s α = .79 for agreement and.87 for induced resolve. This supports the finding of larger interpersonal variance in the agreement with the imageries and a three factor (rather than a two-factor) solution for the agreement scale.

### Comparison of sociodemographic characteristics

There was broad support for imageries about fight against the crisis (*M* = 3.12, *SD* = 0.59) and lessons from the crisis (*M* = 2.68, *SD* = 0.57), while imageries about acceptance of uncontrollability tended to be rejected (*M* = 2.06, *SD* = 0.67). Imageries of challenges tended to lead to a sense of empowerment among participants (*M* = 3.50, *SD* = 0.78), while imagery of humility tended to lead to a sense of helplessness (*M* = 2.70, *SD* = 0.79).

There were significant positive correlations between agreement and age on the factor lessons (*r* = .27, *p* < .001) and acceptance (*r* = .14, *p* < .001), but not fight (*r* = .03, *p* = .324). This means older people agreed more with imagery of lessons and acceptance while there was no age difference for agreement on imagery of fight. Older participants also felt more enabled to deal with the pandemic by imageries of challenges (*r* = .17, *p* < .001) and humility (*r* = .22, *p* < .001).

We also found a significant gender effect, with women (*M* = 2.72, *SD* = 0.49) tending to agree more with the statements about the pandemic compared to men (*M* = 2.65, *SD* = 0.42), with *F*(1, 1.37) = 6.16, *p* = .013, η^2^ = .01. Gender differences were significant for the factor fight (*F* (1, 1.47) = 4.18, *p* = .041, η^2^ <.01; women: *M* = 3.14, *SD* = 0.49; men: *M* = 3.01, *SD* = 0.55) and acceptance (*F*(1, 2.61) = 5.90, *p* = .015, η^2^ < .01; women: *M* = 2.09, *SD* = 0.66; men: *M* = 1.99, *SD* = 0.68), but not lessons (*F*(1, 0.84) = 2.56, *p* = .110, η^2^ < .01; women: *M* = 2.69, *SD* = 0.59; men: *M* = 2.62, *SD* = 0.54). There was no significant effect for induced resolve when comparing women (*M* = 3.15, *SD* = 0.67) and men (*M* = 3.16, *SD* = 0.64), *F*(1, 0.23) = 0.49, *p* = .824, η^2^ < .01.

**Figure 1:**
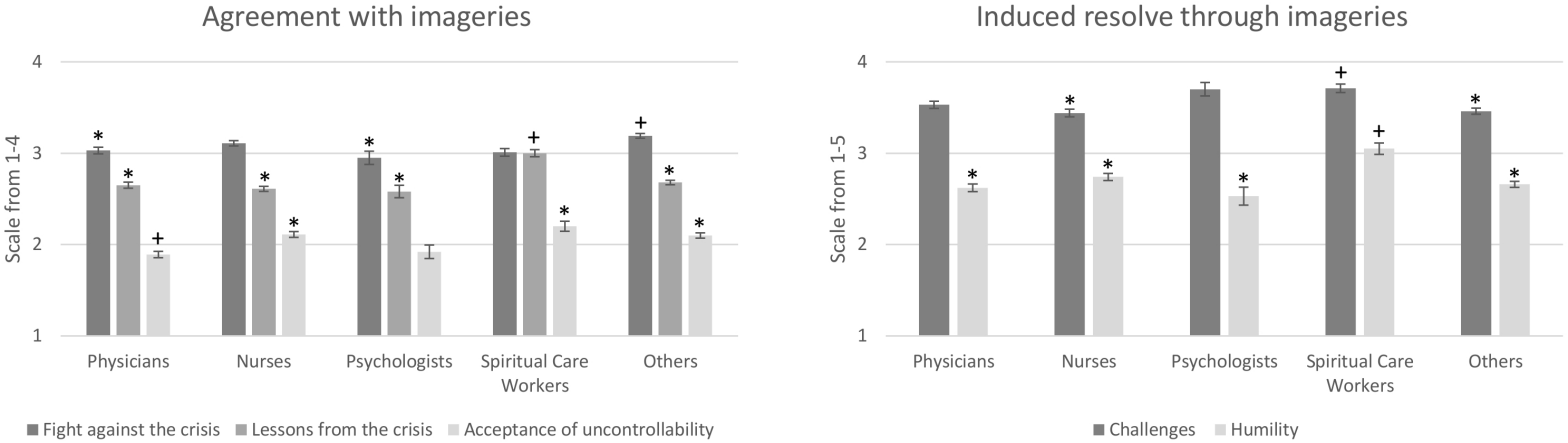
Mean answers with *SEM* on the factors of agreement and induced resolve for different occupational groups. Significant between group differences are marked with * and **^+^**, if no marking is giving, the group doesn’t differ significantly from any group (e.g., for the scale agreement with the factor fight against the crisis, physicians* and psychologists* differ significantly from spiritual care worker**^+^** and others**^+^**, while nurses don’t differ significantly from any group).

With respect to agreement, occupational groups differed (*F*(12, 4395) = 10.61, *p* < .001, η^2^ = .03) on all three factors: fight against the crisis (*F*(4, 1.76) = 5.06, *p* < .001, η^2^ = .01), lessons from the crisis (*F*(4, 4.15) = 12.98, *p* < .001, η^2^ = .03) and acceptance of uncontrollability (*F*(4, 3.33) = 7.63, *p* < .001, η^2^ = .02) (**Figure 1**).

*Post-hoc* analyses revealed that physicians (*M* = 3.03, *SD* = 0.61) and psychologists (*M* = 2.95, *SD* = 0.53) agreed significantly less compared to other occupational groups (*M* = 3.19, *SD* = 0.61) to imageries of fight against the crisis with *d* = .32. We also found that spiritual care workers agreed significantly more (*M* = 3.02, *SD* = 0.43) to lessons learned compared to the other occupations (physicians: *M* = 2.65, *SD* = 0.56; psychologists: *M* = 2.58, *SD* = 0.49; nurses: *M* = 2.61, *SD* = 0.57; others: *M* = 2.68, *SD* = 0.59), with *d* = .74, while physicians agreed significantly less (*M* = 1.89, *SD* = 0.64) to imageries of acceptance compared to nurses (*M* = 2.11, *SD* = 0.66), spiritual care workers (*M* = 2.20, *SD* = 0.59), and others (*M* = 2.09, *SD* = 0.71), with *d* = .45.

For induced resolve, spiritual care workers rated the imageries for the factor challenges to be significantly more helpful (*M* = 3.71, *SD* = 0.51) than nurses (*M* = 3.44, *SD* = 0.84) and others (*M* = 3.46, *SD* = 0.82), with *d* = .34, and for the factor humility to be significantly more helpful (*M* = 3.05, *SD* = 0.67) than other occupations (physicians: *M* = 2.63, *SD* = 0.71; psychologists: *M* = 2.53, *SD* = 0.72; nurses: *M* = 2.74, *SD* = 0.82; others: *M* = 2.66, *SD* = 0.81), with *d* = .55.

### Association of imageries with further parameters

To find out to what extent the imageries are related to protective and vulnerability variables, we computed a linear hierarchical regression model for each factor found in the factor analysis. We included the TPV, IES and optimism as protective variables, and problems with COVID-19 and PHQ-4 as vulnerability variables. The variables were able to significantly predict agreement for the factor fight (*F*(7, 1105) = 15.73, *p* < .001, *R*² = .09), lessons (*F*(7, 1105) = 20.88, *p* < .001, *R*² = .12), and acceptance (*F*(7, 1105) = 5.26, *p* < .001, *R*² = .03) and induced resolve for the factor challenges (*F*(7, 1105) = 24.18, *p* < .001, *R*² = .13) and humility (*F*(7, 1105) = 9.81, *p* < .001, *R*² = .06) (**Table 3**).

**Table 3:** Linear hierarchical regression for the factor agreement and induced resolve.

Independent variables	Dependent variables				
	Agreement with imageries			Induced resolve through imageries	
	1. Fight	2. Lessons	3. Acceptance	1. Challenges	2. Humility
TPV	-.01 (.17)	.12 (.01)***	.07 (.02)***	.06 (.02)**	.07 (.02)**
Problems with COVID-19	.10 (.04)*	-.08 (.03)*	-.02 (.04)	-.17 (.04)***	-.18 (.05)***
PHQ-4	-.06 (.03)	-.05 (.03)	-.01 (.04)	-.13 (.04)	-.04 (.04)
IES	.13 (.03)***	.12 (.03)***	.11 (.04)**	.04 (.04)	-.02 (.04)
Optimism	.03 (.01)*	.04 (.01)**	-.01 (.01)	.08 (.02)***	.03 (.02)

*p* < .05*, *p* < .01**, *p* < .001***.

The relations of the predictors and dependent variables seem to be complex. We found that the prediction of fight imageries is strongly related with the recent experience of trauma (*B* = .13, *p* < .001). Agreeing on imageries of lessons from the crisis adds additionally optimism (*B* = .04, *p* = .004) and transpersonal trust (*B* = .12, *p* < .001). Traumatic experience (*B* = .11, *p* = .006) and transpersonal trust (*B* = .07, *p* < .001) are also good predictors for agreeing on imageries of acceptance. High transpersonal trust is associated with the feeling of being enabled through imageries of challenges (*B* = .06, *p* = .004) and humility (*B* = .07, *p* = .003), while a high number of COVID-19 related problems had a negative association (challenges: *B* = -.17, *p* < .001; humility: *B* = -.18, *p* < .001). Interestingly, anxiety and depression (PHQ-4) did neither predict agreement nor induced resolve through the imageries.

## Discussion

The aim of this study was to analyze imageries of the COVID-19 pandemic in the professional literature and determine its usefulness for different professional groups. Based on the response of a large sample of different groups of professionals in the healthcare sector, we measured the degree of personal agreement with a set of imageries in relation to COVID-19, and whether these imageries could induce a personal resolve to deal with the crisis. Using a factor analysis based on the degree of agreement, we could assign the different imageries to three factors, which we named “fight against the crisis”, “lessons from the crisis” and “acceptance of uncontrollability”. When looking at feelings of empowerment or helplessness associated with the imageries, we found a two-factor structure, with imageries belonging to the first factor having in common that they could be described as “challenges”, whereas imageries of the second factor could be described as expressions of “humility”.

Our findings are in line with previous research that demonstrated a substantial use of metaphors of war, fighting and struggle in our communication about the COVID-19 pandemic (14, 18). We also found that imageries of induced resolve tended to fall into two broader categories, i.e. overcoming obstacles and learning from it versus individual powerlessness in the face of such an immense event. This also expands on previous findings that showed that, while metaphors of war and fighting are the most prevalent (23), they are not necessarily the most helpful, particularly when in the metaphors fighting is more associated with helplessness and uncertainty instead of meaning and sense of mission (16).

In our sample, participants overall agreed more with imageries of fighting and learning, and often disagreed with imageries of acceptance. They also found sentences that represented the crisis as challenge more helpful compared to those that conveyed humility. Age correlated positively with the agreement on all factors but fighting, and with how helpful they found the imageries. It makes sense that life experience comes with a different perspective on such an event, as more crises may have already been mastered in the past. This might lead to a shift away from a heroic perspective of facing a crisis head on towards a perspective of the inevitability of certain consequences independent of how much one fights them, and the chance to grow and learn from difficult situations.

Two professional groups in particular stood out in the group comparison, namely physicians and spiritual care workers. On the one hand, physicians reported the lowest agreement on imageries of acceptance of all groups. This could be attributed to their professional identity, which typically involves a proactive stance against diseases. Physicians are trained not to accept diseases such as infections as something unchangeable over which one has no control and must be accepted. Rather, they learn early on in their training to take responsibility for patients and to regard death as a kind of defeat or failure. Spiritual care workers, on the other hand, stood out given that they reported the highest scores on the scales challenges and humility, suggesting that they found these imageries particularly helpful in comparison. They were also the only group who rated humility as either neutral or positive, while all other groups found images about humility unhelpful. A possible explanation of this finding may be found in the professional understanding of spiritual care workers: While many people consider images that remind them of their own limitations to be frightening and disempowering, it is precisely this experience of facing a seemingly insurmountable challenge with humility and, at the same time, hope that is part of Christian theology (41). Additionally, spiritual care workers are probably working more with imagery on a daily basis and have therefore a better access to them.

Last, we calculated a regression analysis to understand the relationship between the imagery with stress (PHQ-4, IES, problems), transpersonal trust, and optimism. For the agreement to the imageries, the subjective burden in terms of trauma-related psychological symptoms as measured by the IES stood out, which was positively related to the agreement to all categories. This means that agreement with the imageries was particularly high for those feeling currently stressed. It might be that participants, who feel vulnerable and stressed by the pandemic, can relate more to the pandemic associated imagery and are more touched by it. In contrast, regarding the question to what extent the imageries could be helpful for the personal resolve to master the crisis, problems with COVID-19 were negatively related to how helpful one found the images to be. The more problems one had with the crisis, the more helpless one felt due to the imageries. This makes sense when considering that the imageries of the crisis are ultimately a confrontation with the very thing the people are struggling with. Interestingly, the PHQ-4, as a general measure for stress, compared to the IES and problems related to COVID-19, as more specific markers for pandemic related stress, had no correlation with the imagery. This supports the notion that imagery affects specifically people who are emotionally affected by the stressor involved, which in this case is the pandemic.

This is the first study to measure empirically the reaction of health care workers from different occupational groups to imageries of COVID-19, which were excerpted from professional literature. This enabled us to directly compare the impact of the imageries between these groups and map the perception of the language for these groups in terms of agreement with imageries and induced resolve. We also demonstrated that participants who suffered from higher directly COVID-related stress (but not more general depression or anxiety) tended to agree more with the imageries but also felt more helpless through them.

## Limitations

The study is limited in that we only used imageries from scientific literature and surveyed only healthcare professionals. Additionally, the gender imbalance in our sample, with a majority of female participants, further limits the generalizability of our results to broader populations. This gender distribution, while reflective of the workforce in healthcare settings, may not accurately represent other demographic contexts. The study also acknowledges that the majority of our selected literature and imagery comes from Western sources, potentially limiting the applicability of our findings to non-Western contexts and perspectives. This Western focus reflects the current distribution of published research in this area and underscores the need for more diverse cultural research on the topic. We also had to select a number of imageries from a very large body of literature, which is inherently limiting (42).

## Conclusions

Verbal imageries are powerful tools in critical situations. Our study demonstrates that imageries used for the COVID-19 pandemic had differential effects on different professional groups in healthcare in terms of agreement with the imagery used, and in terms of whether it was experienced as enabling for coping with the crisis. On the one hand, this calls for a careful use of imageries when speaking of a crisis. On the other hand, it supports the importance of interprofessional collaboration in healthcare, as the diversity of perspectives (e.g., adding acceptance to a combative spirit) can help to cope with challenges such as experiences of trauma and loss. Furthermore, our study shows how an interdisciplinary cooperation of the humanities (excerpting the imageries) and quantitative psychological research (conducting and evaluating the survey) can represent a genuine enrichment for research. Further studies could explore how and why certain imageries are particularly helpful for certain groups and how an interdisciplinary approach could help in a change of perspective and ultimately make a team more resilient.

## Data availability statement

The raw data supporting the conclusions of this article will be made available by the authors, without undue reservation.

## Ethics statement

The studies involving humans were approved by Ethics Committee of the Medical Faculty of the Rheinische Friedrich-Wilhelms-Universität Bonn. The studies were conducted in accordance with the local legislation and institutional requirements. The participants provided their written informed consent to participate in this study.

## Author contributions

AB: Conceptualization, Data curation, Formal Analysis, Investigation, Methodology, Project administration, Supervision, Writing – original draft, Writing – review & editing. RB: Conceptualization, Data curation, Formal Analysis, Investigation, Methodology, Writing – review & editing. KM: Investigation, Writing – review & editing. ST: Validation, Writing – review & editing. J-KM: Investigation, Writing – review & editing. AC: Writing – review & editing. LR: Conceptualization, Funding acquisition, Methodology, Project administration, Supervision, Writing – review & editing. CR: Conceptualization, Funding acquisition, Investigation, Methodology, Project administration, Supervision, Writing – review & editing. FG: Conceptualization, Funding acquisition, Investigation, Methodology, Project administration, Supervision, Writing – review & editing.
