# Supplementary material for: ‘We are all in the same boat’: a qualitative cross-sectional analysis of COVID-19 pandemic imagery in scientific literature and its use for people working in the German healthcare sector
Source: Front Psychiatry. 2024 Feb 5;15:1296613. doi: 10.3389/fpsyt.2024.1296613 (PMC10875073; doi:10.3389/fpsyt.2024.1296613)
Supplement: Supplementary file 1 [file DataSheet_1.pdf]

Supplement A: Original German imageries used in the study

The heroes and heroines of the crisis are those who stay at their posts and give their all where few see it.	Die Heldinnen und Helden der Krisenzeit sind diejenigen, die auf ihren Posten bleiben und dort, wo es nur wenige sehen, alles geben.
In the pandemic, nurses are on the front lines for all of us.	In der Pandemie stehen die Pflegekräfte für uns alle an vorderster Front.
In times of the pandemic, it becomes clear that we are all in the same boat and can only get ahead if we row together.	In Zeiten der Pandemie wird deutlich, dass wir alle in einem Boot sitzen und nur weiterkommen, wenn wir gemeinsam rudern.
The virus is an invisible enemy.	Das Virus ist ein unsichtbarer Feind.
Now we must seize the opportunity to drive digitization forward.	Jetzt müssen wir die Chance nutzen, die Digitalisierung voranzutreiben.
Our new life begins here and now. Not only after the crisis.	Unser neues Leben beginnt hier und jetzt. Nicht erst nach der Krise.
The pandemic shows that we have to accept that normality means change.	Die Pandemie zeigt, dass wir akzeptieren müssen, dass Normalität Veränderung bedeutet.
The crisis has reminded many people that they too will die.	Die Krise hat viele Menschen daran erinnert, dass auch sie sterben werden.
The pandemic is the stress test for churches to prove that they recognize what people really need.	Die Pandemie ist der Stresstest für die Kirchen, bei dem sie beweisen müssen, dass sie erkennen, was die Menschen wirklich brauchen.
Corona teaches us through distance from each other what closeness really means.	Corona lehrt uns durch die Distanz zueinander, was Nähe wirklich bedeutet.
Such a small virus manages to create a sense of community, we must ensure that the feeling remains.	So einem kleinen Virus gelingt es, ein Gemeinschaftsgefühl zu erzeugen, wir müssen dafür sorgen, dass das Gefühl bleibt.
It is not in our hands how the crisis will turn out, we can only trust.	Es liegt nicht in unserer Hand, wie die Krise ausgeht, wir können nur vertrauen.
Corona shows that there is nothing you can do about the violent storm; you have to endure it patiently.	Corona zeigt, dem heftigen Sturm kann man nichts entgegensetzen, den muss man geduldig aushalten.
Until the pandemic is over, we can only run on sight.	Bis die Pandemie vorbei ist, können wir nur auf Sicht fahren.
